# Updating the classification and routine diagnosis of NSAID hypersensitivity reactions: A WAO Statement

**DOI:** 10.1016/j.waojou.2025.101086

**Published:** 2025-08-12

**Authors:** Antonino Romano, Rocco L. Valluzzi, Emilio Alvarez-Cuesta, Ignacio Ansotegui, Riccardo Asero, Annick Barbaud, Joan Bartra, Sevim Bavbek, Katherine N. Cahill, Pascal Demoly, Inmaculada Doña, María A. Guzmán Meléndez, Mona Kidon, Lily Li, Ricardo Madrigal-Burgaleta, Joanna S. Makowska, Hae-Sim Park, César Picado, Marek Sanak, Masami Taniguchi, Andrew A. White, Steering Committee Authors, Marina Atanaskovic-Markovic, Marina Attanasi, Marcelo Vivolo Aun, Maria Pilar Berges-Gimeno, Lorena Bernal-Rubio, Knut Brockow, Lucrecia Bustamante, Carlo Caffarelli, Yoon-Seok Chang, Manana Chikhladze, Luis Felipe Ensina, Bryan N. Fernandes, Lene Heise Garvey, Pedro Giavina-Bianchi, Eva Gómes, Semanur Kuyucu, Marina Labella, Cristobalina Mayorga, Francesca Mori, Mauro Pagani, Valeria Palma-Pino, Claudio Parisi, Elizabeth Phillips, Elizabeth Powell, Michelle Ramien, Louise Savic, Emilio Solano-Solares, Luciana Kase Tanno, Paula Vazquez-Revuelta, Timothy Watts, Masao Yamaguchi, Review Panel Members

**Affiliations:** a Oasi Research Institute – IRCCS, Troina, Italy & BIOS S.p.A. Società Benefit, Rome, Italy; b Translational Research in Pediatric Specialties Area, Division of Allergy, Bambino Gesù Children's Hospital, IRCCS, Rome, Italy; c Frm CD Allergy Division, Ramon y Cajal University Hospital, Madrid, Spain; d Hospital Quironsalud Bizkaia, Bilbao-Erandio, Spain; e Allergy Department, Clinica San Carlo, Paderno Dugnano, Milan, Italy; f Département de Dermatologie et Allergologie, Sorbonne Université, INSERM, Institut Pierre Louis d’Epidémiologie et de Santé Publique, AP-HP. Sorbonne Université, Hôpital Tenon, Paris, France; g Department of Allergy, Hospital Clinic of Barcelona, University of Barcelona & Institutd’ Investigacions Biomèdiques August Pi i Sunyer (IDIBAPS), RETIC ARADyAL, RICORs REI, Barcelona, Spain; h Department of Chest Diseases, Division of Allergy and Immunology, Faculty of Medicine, Ankara University, Ankara, Turkey; i Department of Medicine, Division of Allergy, Pulmonary, and Critical Care Medicine, Vanderbilt University Medical Center, Nashville, TN, USA; j IDESP, Univ. Montpellier – INSERM, and Department of Pulmonology, Division of Allergy, University Hospital of Montpellier, Montpellier, France; k Allergy Unit and Research Group, Hospital Regional Universitario de Málaga, Instituto de Investigación Biomédica de Málaga y Plataforma en Nanomedicina-IBIMA Plataforma BIONAND, ARADyAL, Málaga, Spain; l Section of Immunology, HIV and Allergy, Department of Medicine, Clinical Hospital University of Chile, Santiago, Chile; m Clinical Immunology, Angioedema and Allergy Unit, Pediatric Allergy Clinic, Safra Children's Hospital, Sheba Medical Center, Tel Hashomer, Israel & Sackler School of Medicine, Tel-Aviv University, Tel Aviv, Israel; n Division of Allergy and Infectious Diseases, Department of Medicine, University of Washington, Seattle, WA, USA; o Allergy & Severe Asthma Service, St Bartholomew's Hospital, Barts Health NHS Trust, London, UK; p Medical University of Lodz, Lodz, Poland; q Department of Allergy and Clinical Immunology, Ajou University School of Medicine, Ajou University Medical Center, Suwon, South Korea; r Pulmonology Department, Hospital Clinic, CIBERES, IDIBAPS, University of Barcelona, Barcelona, Spain; s Jagiellonian University Medical College, Kraków, Poland; t Sagamihara National Hospital, Sagamihara, Japan; u Division of Allergy, Asthma and Immunology, Scripps Clinic, San Diego, CA, USA; v Department of Allergology and Pulmonology, University Children's Hospital, Belgrade, Serbia; w Pediatric Allergy and Pulmonology Unit, Department of Pediatrics, University of Chieti, Chieti, Italy; x Faculdade Israelita de Ciências da Saúde Albert Einstein School of Medicine, Hospital Israelita Albert Einstein, São Paulo, Brazil; y Clinical Immunology and Allergy Division, University of São Paulo School of Medicine, São Paulo, Brazil; z Allergy Division, Ramon y Cajal University Hospital, Madrid, Spain; aa Department of Dermatology and Allergy Biederstein, School of Medicine, Technical University of Munich, Munich, Germany; ab Sección de Alergia., Especialista en Emergencias, Medica Staff – CEA, Hospital Italiano de Buenos Aires Buenos, Aires, Argentina; ac Clinica Pediatrica, Azienda Ospedaliero Universitaria, Department of Medicine and Surgery, University of Parma, Parma, Italy; ad Department of Internal Medicine, Seoul National University Bundang Hospital, Seoul National University College of Medicine, Seongnam, South Korea; ae National Institute of Allergy, Asthma & Clinical Immunology, Associate Professor of the Medical Faculty at Akaki Tsereteli State University. KuTaisi, Tskaltubo, Georgia; af Division of Allergy, Clinical Immunology and Rheumatology, Department of Pediatrics, Federal University of Sao Paulo, Brazil; ag Allergy Clinic, Copenhagen University Hospital Gentofte, Copenhagen, Denmark; ah Department of Clinical Medicine, University of Copenhagen, Copenhagen, Denmark; ai Serviço de Imunoalergologia Centro hospitalar universitário de Santo António Porto, Portugal; aj Department of Pediatric Allergy and Immunology, Mersin University, Faculty of Medicine, Mersin, Turkey; ak Pediatric and Adults Allergy Sections, Italian Hospital of Buenos Aires, Argentina; al Allergy Unit and Research Group, Hospital Regional Universitario de Málaga, Instituto de Investigación Biomédica de Málaga y Plataforma en Nanomedicina-IBIMA Plataforma BIONAND, ARADyAL, Málaga, Spain; am Allergy Unit, Meyer Children's Hospital, IRCCS, Florence, Italy; an Medicine Department, Medicine Ward Mantova Hospital, ASST di Mantova, Mantova, Italy; ao Section of Immunology, HIV and Allergy, Internal Medicine Department, Clinical Hospital University of Chile, Santiago, Chile; ap Allergy and Immunology, Internal Medicine Department, Clínica Alemana Santiago, Chile; aq Department of Medicine & Pathology, Microbiology and Immunology, Vanderbilt University Medical Center, Nashville, TN, USA; ar Children's Allergy Service, Guy's and St. Thomas' NHS Foundation Trust, London, UK; as Department of Pediatrics, University of Calgary, Alberta Children's Hospital, Calgary, Canada; at Department of Medicine, University of Calgary, Calgary, Canada; au Leeds Teaching Hospitals NHS Trust, UK; av Division of Allergy, Department of Pulmonology, Allergy and Thoracic Oncology, University Hospital of Montpellier, Montpellier, France; aw Allergy Department, Hospital Universitari de Bellvitge, Institut d'Investigació Biomèdica de Bellvitge - IDIBELL, Barcelona, Spain; ax Department of Respiratory Medicine & Allergy, Homerton Healthcare NHS Foundation Trust, London, UK; ay Division of Respiratory Medicine, Third Department of Medicine, Teikyo University Chiba Medical Center, Ichihara, Japan

**Keywords:** Drug challenge, NSAID-Induced urticaria/angioedema/anaphylaxis (NIUAA), NSAID-Exacerbated or -induced food allergy (NEFA/NIFA), Patch tests, Skin tests, Update

## Abstract

Hypersensitivity reactions to non-steroidal anti-inflammatory drugs (NSAIDs) have been classified as immediate (or acute) and delayed. Immediate reactions can be further classified into 4 clinical types: NSAID-exacerbated respiratory disease (N-ERD), NSAID-exacerbated cutaneous disease (NECD), NSAID-induced urticaria/angioedema (NIUA), and single NSAID-induced urticaria/angioedema/anaphylaxis (SNIUAA). Specifically, the NIUA type references reactions to ≥2 NSAIDs belonging to different chemical groups, involving urticaria and/or angioedema in patients with no underlying chronic spontaneous urticaria. However, there are patients meeting cross-reactive criteria for NIUA phenotype who report reactions that involve 2 organ systems (eg, cutaneous and respiratory; cutaneous and gastrointestinal) and have been termed “blended”. In pediatrics, this type of reaction is recognized and has been termed NSAID-induced urticaria/angioedema/anaphylaxis (NIUAA), an acronym we suggest be extended now to adults. There are small subgroups of N-ERD patients who also report skin symptoms and, alternatively, NECD patients who report respiratory symptoms. These 2 subgroups could be diagnosed as having mixed N-ERD and mixed NECD, respectively. In fact, they are patients suffering from N-ERD or NECD who have had reactions consistent with anaphylaxis.

In the current classifications of NSAID hypersensitivity, the reactions in which NSAIDs act as aggravating factors or cofactors in subjects with sensitization to foods are not included. Recently, this type of reactions has been defined as NSAID-exacerbated food allergy (NEFA) and NSAID-induced food allergy (NIFA), respectively.

This Statement of the World Allergy Organization (WAO) aims to update both the classification of hypersensitivity reactions to NSAIDs and their diagnosis, addressing the novel issues.

## Introduction

Based on the chronological criterion, European^1^ and North American^2^^,^^3^ guidelines have classified hypersensitivity reactions to non-steroidal anti-inflammatory drugs (NSAIDs) as immediate (or acute) and delayed. Immediate reactions occur within hours (≤6 h^2^^,^^3^ or ≤ 24 h^1^) of exposure, whereas, delayed reactions occur >6 h^2^^,^^3^ or > 24 h. These guidelines^1^, ^2^, ^3^ have further classified immediate/acute reactions to NSAIDs as 4 clinical types: aspirin/NSAID-exacerbated respiratory disease (AERD/N-ERD), NSAID-exacerbated cutaneous disease (NECD), NSAID-induced urticaria/angioedema (NIUA), and single NSAID-induced urticaria/angioedema/anaphylaxis (SNIUAA). Specifically, the NIUA type refers to reactions to ≥2 NSAIDs belonging to different chemical groups (ie, chemically unrelated, Table 1), which are characterized by urticaria and/or angioedema in patients with no underlying chronic spontaneous urticaria (CSU). However, some patients meeting cross-reactive criteria for NIUA phenotypes may present with reactions involving 2 organ systems (eg, cutaneous and respiratory; cutaneous and gastrointestinal) in the absence of underlying CSU, asthma, or sinus disease. Such reactions have been termed "blended",^4^, ^5^, ^6^, ^7^, ^8^, ^9^ as in an earlier classification by Stevenson et al.^10^ According to Sampson et al,^11^ however, these hypersensitivity reactions should be considered anaphylactic, as in some studies^12^ and reviews^13^^,^^14^ regarding immediate reactions to NSAIDs. Indeed, they were defined by Stevenson et al^10^ themselves as "mild" anaphylactic reactions. In pediatrics, this type of reaction is recognized and has been termed NSAID-induced urticaria/angioedema/anaphylaxis (NIUAA),^15^ an acronym we suggest be extended now to adults and which we will use instead of NIUA in this article. Moreover, there are studies in which N-ERD patients also report skin symptoms and NECD patients report respiratory symptoms.^16^, ^17^, ^18^, ^19^, ^20^, ^21^, ^22^ In one of these studies,^16^ the adjective "blended" has again been used to define anaphylactic reactions induced by multiple NSAIDs in patients without underlying CSU, asthma, or sinus disease and hypersensitivity reactions to ≥2 chemically unrelated NSAIDs in patients with both asthma/chronic rhinosinusitis and CSU.

**Table 1:** Non-steroidal anti-inflammatory drugs (NSAIDs) classified according to the chemical structure.

Chemical group	Drug
Salicylic acid derivatives	Acetylsalicylic acid (aspirin), sodium salicylate, salsalate, diflunisal, sulfasalazine
Propionic acid derivatives	Dexibuprofen, dexketoprofen, fenoprofen, flurbiprofen, ibuprofen, ketoprofen, naproxen, piketoprofen
Acetic acid derivatives	Aceclofenac, diclofenac, etodolac, fentiazac, ketorolac, indomethacin, sulindac, tolmetin
Anthranilic acid derivatives (Fenamates)	Etofenamate, flufenamic acid, meclofenamic acid, mefenamic acid, niflumic acid, tolfenamic acid
Pyrazolone derivatives	Aminophenazone, dipyrone (metamizole, noramidopyrine), phenylbutazone, propyphenazone
Enolic acid derivatives (oxicams)	Lornoxicam, meloxicam^a^, piroxicam, tenoxicam
Para-aminophenol derivatives	Paracetamol (Acetaminophen)^c^
Sulphonanilide derivatives	Nimesulide^a^
Coxibs^b^	Celecoxib, etoricoxib, parecoxib

a Meloxicam and nimesulide are preferential COX-2 inhibitors.

b Selective COX-2 inhibitors.

c Paracetamol is often included into the category of NSAIDs; however, it has little anti-inflammatory activity and COX-1 inhibition is only noted at cumulative doses >4 g per day.

Of note, in the current European^1^ and North American classifications,^2^^,^^3^ the reactions in which NSAIDs act as aggravating factors or cofactors in subjects with sensitization to foods are not included. In 2 recent European prospective studies,^23^^,^^24^ these types of reactions were defined as food-dependent NSAID-induced hypersensitivity (FDNIH) and NSAID-exacerbated or NSAID-induced food allergy (NEFA/NIFA), respectively. In these 2 studies,^23^^,^^24^ approximately 16% and 18% of 328 and 414 patients with immediate reactions to NSAIDs experienced FDNIH and NEFA/NIFA reactions, respectively.

This Statement of the World Allergy Organization (WAO) aims to update both the classification of hypersensitivity reactions to NSAIDs and their diagnosis, addressing the novel issues.

## N-ERD

### Clinical presentation

#### Classical N-ERD

N-ERD is characterized by the triad of asthma, chronic rhinosinusitis with nasal polyps (CRSwNP), and respiratory reactions to cyclooxygenase (COX)-1 inhibitors. Each component of the triad can develop over a span of months to many years. Peak incidence is in the third and fourth decade of life, with cases reported as early as puberty onset and as late as the eighth decade of life.^25^ Most patients with N-ERD have comorbid CRSwNP and olfactory disturbances, which often precede asthma symptoms.^25^, ^26^, ^27^ The respiratory reactions to COX-1 inhibitors involve the acute onset of nasal congestion, rhinorrhea, sneezing, nasal pruritus, ocular chemosis, and/or bronchospasm within 30 min to 3 h of exposure. These respiratory reactions differentiate N-ERD from NSAID-tolerant asthma and/or CRSwNP.

#### N-ERD variants

The spectrum of clinical presentations of N-ERD is broad. Due to the range of the underlying asthma and CRSwNP severity and the time delay between onset of the first symptoms of N-ERD and the development of all 3 components of the clinical triad, partial presentations of N-ERD may be observed in the clinic. The development of respiratory reactions to COX-1 inhibitors in patients with underlying respiratory disease is the minimum requirement for the diagnosis of N-ERD. A significant subgroup (∼20%) of patients with N-ERD can develop extra-respiratory signs and symptoms with exposure to COX-1 inhibitors.^17^^,^^20^ Extra-respiratory symptoms can include a pruritic macular cutaneous eruption, urticaria, angioedema, angina-like chest pain, abdominal pain, nausea, vomiting, and diarrhea.^17^^,^^21^^,^^28^ In the clinical experience of the expert panel, a small subgroup of patients with N-ERD may present with cutaneous manifestations including macular eruptions that are present in the absence of a COX-1 inhibitor exposure. N-ERD patients with chronic macular eruptions, urticaria and/or angioedema, or COX-1 inhibitor-induced extra-respiratory manifestations are subgroups which could be diagnosed as having mixed N-ERD.

#### Prevalence

Prevalence of N-ERD in population-based studies was 0.1%–0.6%.^29^, ^30^, ^31^ According to a recent meta-analysis, prevalence is 7.2% among adults with asthma, and up to 14.9% among those with severe asthma.^32^

### Pathogenic mechanism

Disturbances in the COX and 5-lipoxygenase (5-LO) pathways of arachidonic acid metabolism characterize N-ERD.^33^^,^^34^ Constitutive COX-1 and inducible COX-2 initiate the synthesis of prostaglandin E_2_ (PGE_2_) and PGD_2_.^34^ PGE_2_ exerts anti-inflammatory effects while PGD_2_ is a potent bronchoconstrictor and pro-inflammatory mediator produced mainly by mast cells.^17^^,^^34^^,^^35^

The inhibition of COX-1 by NSAIDs, and high-dose paracetamol (acetaminophen) in a minority of patients, precipitates respiratory tract reactions.^36^ The increased sensitivity of these patients to NSAIDs appears to be associated with down-regulated expression of COX-2 and microsomal PGE_2_ synthase 1 and reduced production of protective PGE_2_.^37^, ^38^, ^39^, ^40^ Diminished expression of the anti-inflammatory EP2 receptor of PGE_2_ on inflammatory and stromal cells contributes to the functional deficiency of PGE_2_.^41^, ^42^, ^43^, ^44^

Arachidonic acid is converted by 5-LO to leukotriene A_4_ (LTA_4_), which is subsequently converted to LTD_4_ and LTE_4_ that are excreted in the urine.^45^ Cysteinyl LTs (CysLTs) differentially stimulate 3 cysLT receptors: CysLT_1_R (LTD_4_), CysLT_2_R (LTC_4_), and CysLT_3_R/GPR99 (LTE_4_) contracting bronchial smooth muscles, increasing vascular permeability, and activating inflammatory cell influx.^45^

N-ERD patients have elevated levels of cysLTs which result from the increased expression of 5-LO and LTC_4_ synthase in the airways^46^, ^47^, ^48^ and increased platelet adherence to granulocytes which potentiate cysLT production from granulocytes.^49^^,^^50^ Inhibition of COX-1 activity in patients with N-ERD further reduces the “braking” effects of PGE_2_/EP2 on mast cells and 5-LO activity, which increases the release of cysLTs in urine and airway fluids, precipitating respiratory symptoms.^46^ PGE_2_ inhibits LO-activity,^51^ preventing clinical symptoms and the increased cysLT release in response to acetylsalicylic acid (ASA)—commonly known under the trade name of “aspirin”—in N-ERD patients.^52^ By mechanisms still unknown, patients with N-ERD almost always tolerate selective COX-2 inhibitors during observed drug challenges (DCs).^53^

### Diagnosis

#### Clinical history and exam

**Fig. 1:**
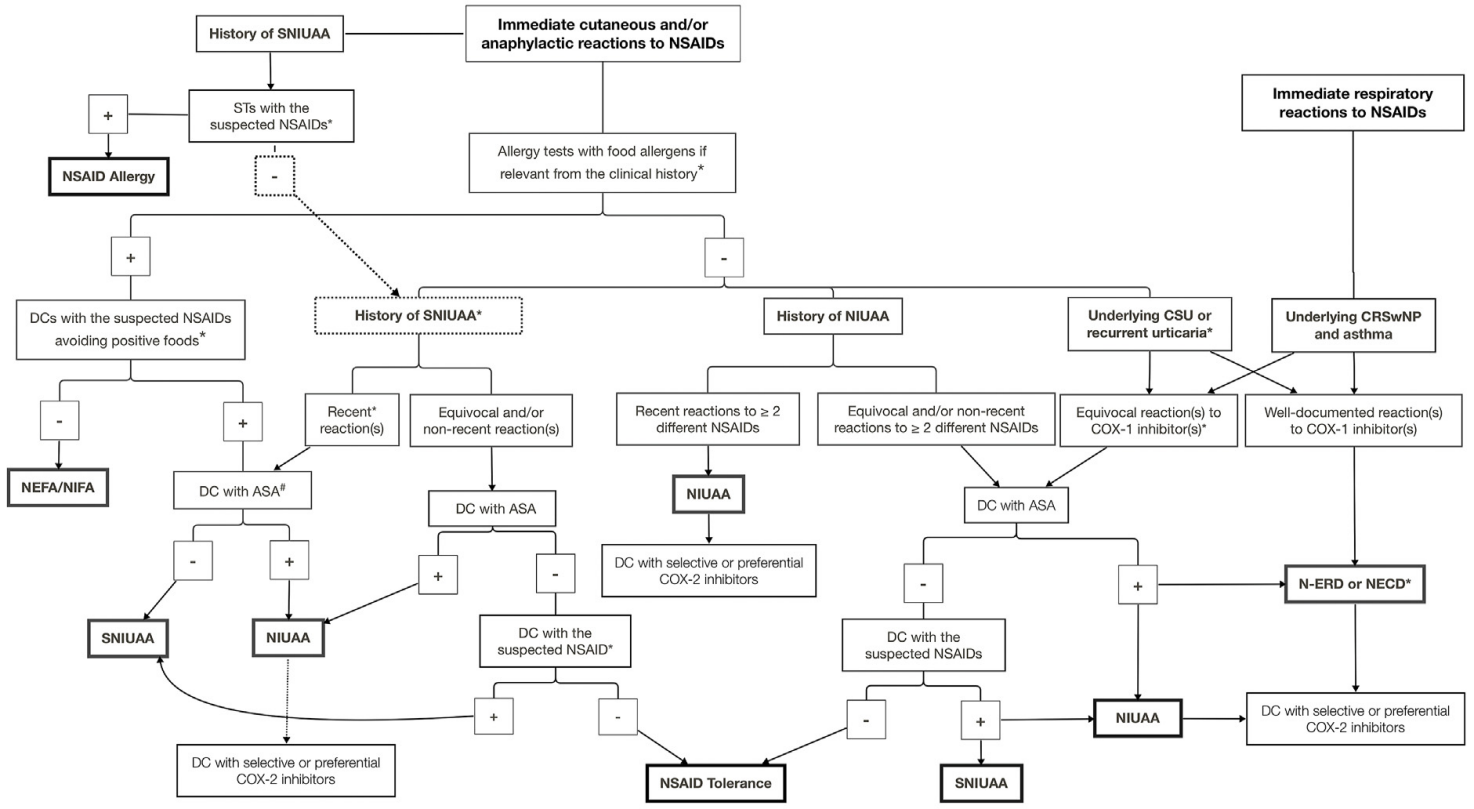
Diagnostic algorithm for patients with histories of immediate reactions to non-steroidal anti-inflammatory drugs ASA, acetylsalicylic acid; COX-1, cyclooxygenase-1; CRSwNP, chronic rhinosinusitis with nasal polyps; CSU, chronic spontaneous urticaria; DC, drug challenge; NECD, NSAID-exacerbated cutaneous disease; NEFA, NSAID-exacerbated food allergy; N-ERD, NSAID-exacerbated respiratory disease; NIFA, NSAID-induced food allergy; NIUAA, NSAID-induced urticaria/angioedema and/or anaphylaxis; NSAID, non-steroidal anti-inflammatory drug; SNIUAA, single NSAID-induced urticaria/angioedema or anaphylaxis; SPTs, skin prick tests; STs, skin tests. ∗See text. ^#^ or with another potent COX-1 inhibitor (e.g., ibuprofen or ketoprofen) if ASA is the suspected drug

A clinical diagnosis of N-ERD can often be established by history and physical exam alone.^54^^,^^55^ Fig. 1 shows the diagnostic algorithm for patients with histories of immediate reactions to NSAIDs. Clinicians should ascertain the signs and symptoms of each component of the clinical triad: asthma, CRSwNP, and respiratory reactions to COX-1 inhibitors.

*Asthma* – The diagnosis requires a history of symptoms including cough, shortness of breath, wheeze, and/or chest tightness and evidence of variable airflow obstruction and/or airway hyperresponsiveness.^56^ Exam findings of expiratory wheeze and hyperinflation are supportive.

*CRSwNP* – The diagnosis requires a history of symptoms including persistent nasal obstruction, anosmia, and anterior or posterior discharge. Anterior rhinoscopy is often sufficient to visualize bilateral nasal polyps in N-ERD; nasal endoscopy should be performed in those with symptoms consistent with nasal polyposis not visualized on anterior rhinoscopy exam. Young age at time of nasal polyp resection, rapid regrowth of nasal polyps following surgical resection, and high medication needs to treat CRSwNP should increase the clinical suspicion for a N-ERD diagnosis.^57^

*COX-1 inhibitor hypersensitivity* – The diagnosis requires the development of acute upper and/or lower respiratory symptoms (nasal congestion, rhinorrhea, sneezing, conjunctival irritation, ocular chemosis, pruritus, bronchospasm) following ingestion of a therapeutic dose of a COX-1 inhibitor.^54^^,^^55^ A history of respiratory reactions triggered by more than one COX-1 inhibitor confirms the NSAID cross-reactivity observed in N-ERD.

When a component of the triad is in question, additional diagnostic evaluation is appropriate.

#### Diagnostic data

Diagnostic testing supports the diagnosis of asthma and CRSwNP. Oral ASA challenge testing is the gold standard for diagnosing hypersensitivity to NSAIDs, and sensitive and specific for N-ERD.^54^

*Lung function assessments* – Standard diagnostic evaluation for asthma is appropriate in N-ERD if an asthma diagnosis has not been established. Spirometry with bronchodilator response testing or peak flow monitoring to capture variable airflow obstruction should be the first step. Rarely, airway hyperresponsiveness testing may be considered. Limited data exist on the utility of methacholine or mannitol testing in N-ERD to distinguish N-ERD from NSAID-tolerant asthma.^58^

*Laboratory data* – Peripheral blood eosinophilia (>300 cells/μL) and elevated exhaled breath nitric oxide levels (>25 ppb) are common in N-ERD but are neither sensitive nor specific for the diagnosis of N-ERD.^57^ Additional measurement of urinary LTE4 levels is useful, but not sufficient, for the diagnosis of N-ERD.^59^

*Imaging data* – Sinus computed tomography (CT) scans may be reserved for those requiring surgery. Other imaging modalities are not recommended. A diagnosis of nasal polyposis is best established on nasal endoscopy.

*Histopathology* – Tissue excised during sinonasal surgery should be evaluated and typically shows high numbers of eosinophils/high power magnification.^60^ Nasal polyp biopsy is not recommended in the diagnostic evaluation.

*Challenge Testing* – Up to 15% of patients with N-ERD may not report a clinical history consistent with COX-1 inhibitor hypersensitivity.^26^ Additionally, some patients present with equivocal clinical histories. In these circumstances, a diagnostic oral DC with ASA is used to establish an N-ERD diagnosis. In effect, it has the highest sensitivity (80–90%).^61^ Additional options, where available, include bronchial (inhalation) challenge with soluble lysine acetylsalicylate^61^^,^^62^ and nasal challenge with soluble lysine acetylsalicylate^61^^,^^63^^,^^64^ or ketorolac.^65^ These inhaled lysine acetylsalicylate or ketorolac challenges are less sensitive (77–90%) but safer than the oral ASA challenge.^61^^,^^62^^,^^65^ If the inhaled DC yields a negative result, an oral DC should be performed. Patients undergoing oral or inhaled DC should be in a stable condition, with an FEV1 ≥ 70% of predicted value.^66^ Current treatment with β-blockers is contraindicated in patients referred to the DC.^2^^,^^54^ The latter, independent of route, involves ASA/ketorolac exposure, most often with dose escalation, and monitoring for the development of acute upper and/or lower respiratory symptoms. Tolerance of 325 mg of ASA during a graded DC without upper or lower reaction symptoms generally excludes COX-1 inhibitor hypersensitivity.^54^ However, a negative DC does not fully exclude hypersensitivity, especially if the patient has well-controlled asthma, underwent recent surgical excision of nasal polyps, or is taking leukotriene (LT) receptor antagonists or high-dose systemic corticosteroids, factors associated with reduced reaction severity and negative reactions.^67^, ^68^, ^69^, ^70^, ^71^, ^72^ Of note, in a study by White et al concerning 676 patients with suspected AERD who had undergone oral aspirin challenges,^70^ the effect of pretreatment with controller medications (ie, long-acting β2-agonists, systemic corticosteroids, inhaled corticosteroids, and LT modifier drugs) on the outcome of such challenges was measured. Only pretreatment with LT modifier drugs enhanced the safety of oral aspirin challenges by significantly decreasing the degree of asthmatic responses.

No *in vitro* tests are sensitive or reliable to be recommended in the diagnosis of N-ERD.

## NECD

### Clinical presentation

It has long been known that up to 30% of patients with CSU, may experience a marked, acute, and short-lived exacerbation of their disease, including angioedema, within minutes to hours after taking chemically unrelated NSAIDs.^6^ This condition, which rarely progresses to anaphylaxis, is termed NECD.^1^, ^2^, ^3^ There is a small subgroup of patients with NECD who also report respiratory symptoms (ie, anaphylaxis) after taking NSAIDs, and who may be classified as having mixed NECD (Table 2).

**Table 2:** Classification of NSAID hypersensitivity reactions

Phenotype	Clinical manifestation	Chronology^a^	Underlying disease	Pathogenic Mechanism	Cross-reactive phenotype
**NSAID-exacerbated respiratory disease (N-ERD)**	*Classical variant*: Nasal airway congestion, rhinorrhea, sneezing, nasal pruritus, ocular chemosis, and/or bronchospasm *Mixed variant:* Classical plus cutaneous or other non-respiratory symptoms	**Immediate** (≤6 h)	**Chronic rhinosinusitis with nasal polyps and/or asthma**	**COX-1 inhibition**	**Yes**
**NSAID-exacerbated cutaneous disease (NECD)**	*Classical variant:* Urticaria and/or angioedema *Mixed variant:* Classical plus respiratory symptoms	**Immediate** (≤6 h)	**Chronic spontaneous urticaria or recurrent urticaria**	**COX-1 inhibition**	**Yes**
**NSAID-induced urticaria/angioedema/anaphylaxis (NIUAA)**	Urticaria, angioedema, anaphylaxis	**Immediate** (≤6 h)	None	**COX-1 inhibition**	**Yes**
**Single-NSAID-induced urticaria/angioedema/anaphylaxis (SNIUAA)**	Urticaria, angioedema, anaphylaxis	**Immediate** (≤6 h)	None	**IgE-mediated**	**No**
**NSAID-exacerbated/induced food allergy (NEFA/NIFA)**	Urticaria, angioedema, anaphylaxis	**Immediate** (≤6 h)	**Food hypersensitivity/sensitization**	**NSAID acts as aggravating factor (NEFA) or cofactor (NIFA)**^b^	
**Single-NSAID-induced delayed hypersensitivity reactions (SNIDHRs)**	CD, FDE, MDE, PACD, SDRIFE, SCARs (e.g., AGEP, DIHS/DRESS, GBFDE, SJS/TEN), systemic (allergic) CD, vasculitis	**Delayed** (>6 h)	None	**T-cell-mediated**	**No**

AGEP, acute generalized exanthematous pustulosis; CD, contact dermatitis; COX, cyclooxygenase; DIHS, drug-induced hypersensitivity syndrome; DRESS, drug reaction with eosinophilia and systemic symptoms; e.g., for example; FDE, fixed drug eruption; GBFDE, generalized bullous fixed drug eruption; h, hour(s); MDE, morbilliform drug eruption; NSAID, non-steroidal anti-inflammatory drug; PACD, photoallergic contact dermatitis; SCARs, severe cutaneous adverse reactions; SDRIFE, symmetrical drug-related intertriginous and flexural exanthema; SJS, SSLR, serum sickness like reaction; Stevens-Johnson syndrome; TEN, toxic epidermal necrolysis.

a Time interval between NSAID exposure and onset of symptoms.

b See text.

In NECD, NSAID hypersensitivity does not seem to depend on the endotype of CSU, as its distribution is identical in early-, slow- or non-responders to the anti-IgE monoclonal antibody, omalizumab,^73^ nor depends on the presence or absence of autoreactivity.^74^ The intensity of the reaction to NSAIDs depends on the activity of the underlying CSU and patients can tolerate NSAIDs during periods of CSU remission.^75^

### Pathogenic mechanism

NECD shows some typical features that make it unique in the panorama of hypersensitivity to NSAIDs. In fact, reactions are clearly associated with the COX-1 inhibiting activity of the offending drugs, as patients generally tolerate weak COX-1 inhibitors (eg, paracetamol) and preferential (eg, meloxicam and nimesulide) or selective COX-2 inhibitors (ie, coxibs).^1^^,^^6^ This suggests that the pathogenic mechanism of NECD is identical or very similar to that viable in N-ERD and in most NIUAA patients.^1^ An indirect confirmation of such pathogenic mechanism comes from the observation that, in some cases, LT receptor antagonists were able to prevent NSAID-induced exacerbations in NECD.^76^ Further, as the intensity of the reaction induced by NSAIDs parallels the activity of the underlying disease,^75^^,^^77^ patients may tolerate NSAIDs during periods in which the disease is in spontaneous remission or controlled by omalizumab^78^ or antihistamines.^79^ Altogether, NECD and NIUAA patients are clinically indistinguishable,^80^ and there are data suggesting that both SNIUAA and NIUAA patients may evolve into a NECD (or in other words, that NSAID hypersensitivity might precede the occurrence of spontaneous urticaria), although this is controversial.^80^, ^81^, ^82^, ^83^ Further, NECD and NIUAA show the same eicosanoid mediator profile both at baseline and following ASA challenge.^84^ The reasons for the rather frequent association between CSU and NSAID hypersensitivity are unclear. It is possible to hypothesize that CSU (notably, the autoimmune or autoallergic form) is genetically linked to the changes in eicosanoid metabolism that also characterize patients with NIUAA. It is also possible that these patients show a specific genetic profile although genetic studies have been mainly focused on N-ERD.^85^

### Diagnosis

In patients with NECD, it is not advisable to perform a graded DC with ASA in active CSU^1^ or recurrent urticaria and well-documented (ie, confirmed by medical records or pictures in patients’ dossiers/smartphones) hypersensitivity reactions to ≥ 2 COX-1 inhibitors belonging to different chemical groups.^86^ Indeed, patients with uncontrolled urticaria are unlikely to tolerate these NSAIDs at any dose.^3^ A history of cutaneous reactions triggered by ≥ 2 chemically unrelated COX-1 inhibitors confirms the NSAID cross-reactivity observed in NECD.^1^

Potential biomarkers such as LTE_4_ and the ratio LTE_4_/9a,11b-PGF_2_, and serum tryptase have been proposed as *in vitro* diagnostic tests but data are limited.^6^

## NIUAA

### Clinical presentation/Demographics

NIUAA is the most common NSAID hypersensitivity category for all age groups, including children, representing more than 40% of all hypersensitivity reactions to NSAIDs in adults,^87^ and between 45% and 60% in the pediatric population.^88^^,^^89^ This entity is more common in adult females (60–65%)^5^^,^^87^^,^^90^^,^^91^ while in the pediatric population there is a male predominance (70%).^15^^,^^88^^,^^92^ Contrary to NECD, NIUAA patients do not have underlying CSU,^1^ although controversies exist about whether NIUAA can precede the appearance of CSU.^81^^,^^83^ Some reports suggest that up to one third of patients with an initial diagnosis of NIUAA may develop CSU over time, although other prospective studies have observed a similar rate for these patients compared to the general population.^80^^,^^81^^,^^83^

Up to 60% of NIUAA cases are atopic, most commonly sensitized to dust mite, making this an important difference from other NSAID hypersensitivity phenotypes.^5^^,^^87^^,^^89^, ^90^, ^91^^,^^93^ In the first/second decade of life, some patients with respiratory symptoms due to house dust mite sensitization may develop periorbital angioedema after NSAID intake.^90^^,^^91^^,^^93^^,^^94^ These cases underscore the potential importance of dust mite sensitization in the mechanism of some of these NSAID reactions.

Symptoms are typically elicited by strong COX-1 inhibitors within hours of drug ingestion, but high doses of weak COX-1 inhibitors (e.g., ≥1 g of paracetamol) can also induce symptoms in up to 20% of adult patients.^87^^,^^95^

Most patients with cross-reactive NSAID hypersensitivity report isolated cutaneous symptoms involving urticaria and/or angioedema. However, between 9% and 28% of adults^5^^,^^16^^,^^87^^,^^96^ and up to 40% of children^92^ may report reactions consisting of blended features or anaphylaxis. The term “blended” refers to symptoms/signs that extend outside of the skin and have characteristics consistent with mild anaphylaxis. Within the subgroup of individuals who develop reactions involving more than one organ system, cutaneous symptoms generally appear first. A combination of cutaneous and respiratory symptoms is the most frequent presentation, followed by cutaneous and gastrointestinal symptoms; involvement of cutaneous, respiratory, and gastrointestinal features, or respiratory and gastrointestinal symptoms without cutaneous finding are much less common.^5^ We suggest using NIUAA to classify all “blended” reactions and those previously classified as NIUA.

### Pathogenic mechanism

As with other cross-reactive phenotypes, including NECD and N-ERD, reactions consistent with NIUAA are thought to occur due to inhibition of COX-1 leading to aberrant generation of LTs and downstream release of mast-cell- and eosinophil-derived mediators. Baseline levels of urinary LTE_4_ and PGE_2_, and PGD_2_ metabolites are similar for patients with NIUAA, NECD and SNIUAA, whereas levels of LTE_4_ and 9α,11β-PGF_2_ significantly rise only for those with NIUAA and NECD following ASA administration.^84^ Notably, the increase in LTE_4_ following ASA administration has been found to be higher for patients with N-ERD with mixed features and NIUAA, compared to patients with NECD or NIUAA with cutaneous features alone.^8^ In the pathophysiology of this phenotype of NSAID hypersensitivity there is no production of specific IgE against the responsible drugs, but sensitization to aeroallergens seems to play a role.^5^^,^^22^^,^^87^^,^^89^, ^90^, ^91^^,^^93^ However, unlike in patients with mite sensitization and isolated periorbital angioedema induced by multiple NSAIDs,^90^ in other patients with NIUAA, the role of aeroallergen sensitization has not yet been well established.

### Diagnosis

#### Basophil activation test (BAT)

The BAT test using flow cytometry technology has been used to evaluate NIUAA patients, although its utility is unclear given the lack of consistent results.^97^, ^98^, ^99^, ^100^ In the only study that exclusively included NIUAA patients, the sensitivity of BAT was high, but the very low specificity limited its utility for such patient.^101^ A recent European position paper^102^ recommends against the use of BAT for the evaluation of cross-reactive types of NSAID hypersensitivity, including NIUAA.

#### Oral challenge protocols

Oral DCs represent the gold standard for proving NSAID hypersensitivity. According to most of the authors of this paper, however, patients without CSU who report recent (ie, that occurred <5 years before allergy testing) and well-documented urticarial/angioedematous/anaphylactic reactions to ≥ 2 chemically unrelated NSAIDs can be diagnosed as having NIUAA although this criterion could lead to an overdiagnosis of NIUAA. In fact, in a Spanish study,^103^ the ASA challenge was positive in 63% of patients who had reacted to 2 different NSAIDs, while it was positive in 92% of those with reactions to ≥3 chemically unrelated NSAIDs. In any case, we recommend that patients with equivocal/doubtful or non-recent reactions (i.e., that occurred >5 years before allergy testing) to ≥ 2 different NSAIDs undergo DCs with ASA.^1^ Those who react can be diagnosed with NIUAA. Patients who tolerate ASA challenges should undergo DCs with suspected NSAIDs. This diagnostic modality represents the best way to discriminate between NIUAA and SNIUAA, as well as to exclude NSAID hypersensitivity (Fig. 1). Some studies demonstrated that the total number of DCs per patient decreases if ASA DC is performed first, followed by additional DCs as needed.^104^^,^^105^ However, some authors favor DCs with the suspected NSAIDs in order to confirm the diagnosis and quickly find an alternative NSAID.^106^ Of note, due to some concerns regarding the side effects of ASA, particularly Reye's syndrome, most European pediatric allergists do not perform ASA challenges to classify their patients with NSAID hypersensitivity reactions.^107^ Most DC protocols consider starting with a low dose (ie, one-tenth or one-quarter of the therapeutic dose). In a study using a two-step protocol,^96^ most reactions to the second step occurred after 60 min; therefore, a longer observation after the second dose (up to 120 min) was recommended.

### Area of uncertainty

An area of uncertainty regards patients with reactions to multiple, but not all COX-1 inhibitors. Subjects reacting to several NSAIDs are usually considered cross-hypersensitive, whereas those responding to only one NSAID with good tolerance of other chemically unrelated NSAIDs are considered selective responders.^1^ However, several cases of patients who had reactions with several chemically unrelated NSAIDs but tolerated ASA have been reported.^108^, ^109^, ^110^, ^111^ In these cases, the tolerance of high doses of ASA (>500 mg) rules out the possibility of an underlying pharmacological dose-dependent mechanism related to COX-1 inhibition.^112^ According to Blanca-López et al,^113^ patients with reactions induced by multiple NSAIDs should be tested for tolerance of ASA; if it is tolerated, the diagnosis of NSAID-multiple selective immediate reaction (NMSIR)^6^ must be considered. It is unclear how frequently this phenomenon occurs, and if it is in fact IgE mediated, why these patients might develop an IgE-mediated response to several chemically unrelated NSAIDs but not to other drugs commonly associated with IgE mechanisms such as β-lactams. However, carrying out challenges with ASA in these patients involves a significant risk of reactions. Therefore, we suggest reserving this approach only for patients with an indication for aspirin therapy. As far as we know, no biomarkers for this condition have been detected so far.

### Management of patients with cross-reactive types of immediate NSAID hypersensitivity: administration of alternative agents

Selective (ie, rofecoxib, celecoxib, parecoxib, valdecoxib, and etoricoxib) or preferential (eg, meloxicam and nimesulide) COX-2 inhibitors are relatively safe alternatives in patients with N-ERD, NECD, or NIUAA.^53^^,^^114^, ^115^, ^116^, ^117^, ^118^ A meta-analysis^115^ of all blinded, placebo-controlled clinical trials published on or before April 11, 2013 (in total 14) evaluated the effects of these alternative agents in patients with N-ERD. Compared with placebo, no significant difference in adverse respiratory effects after acute exposure to selective COX-2 inhibitors was found. Instead, there was a small but statistically significant risk with meloxicam and nimesulide. Indeed, reactions to preferential COX-2 inhibitors occurred in approximately 1 in 13 patients. Subsequently, a systematic search of MEDLINE databases^53^ identified 62 studies published before November 2018 involving patients with any type of NSAID hypersensitivity who underwent a total of 3218 single- or double-blinded placebo-controlled DCs with selective COX-2 inhibitors, 106 (3.3%) of which caused reactions. The rate of DCs positive to celecoxib, etoricoxib, or parecoxib (the only coxibs still available) was 3.5% (40 of 1140 DCs), 5.4% (32 of 595), and 0% (0 of 116), respectively. Notably, 67 (93.1%) of the 72 reactions consisted of urticaria/angioedema, in 2 cases accompanied by respiratory symptoms. Moreover, the results of 753 DCs (297 with celecoxib, 356 with rofecoxib, 88 with etoricoxib, and 12 with parecoxib) performed in patients with NSAID-induced respiratory reactions were analyzed separately; only 1 DC (0.13%) was positive (to rofecoxib). This study^53^ also found that cross-reactivity was higher with meloxicam than with the coxibs, with a reaction rate of 4.7% among 847 single-blind placebo-controlled DCs. However, in a study^119^ published in 2013 and not included in the above meta-analysis,^115^ 3 (2.9%] of 104 patients with N-ERD reacted to celecoxib DCs. Interestingly, most patients with cross-reactive types of NSAID hypersensitivity who react to a selective COX-2 inhibitor may tolerate another one^120^^,^^121^ possibly related to the higher COX-2 selectivity of etoricoxib compared to celecoxib.^122^

Regarding preferential COX-2 inhibitors, in a retrospective study^114^ that was not included in the above systematic review,^53^ single-blinded placebo-controlled DCs with nimesulide and meloxicam caused reactions in 11 (8.1%) of 112 and 6 (9.8%) of 74 cross-hypersensitive patients, respectively*.* Considering blinded, placebo-controlled clinical trials published after 2019,^116^, ^117^, ^118^ the highest rate of cross-reactivity with celecoxib, meloxicam, and nimesulide was found in 49 N-ERD patients, 4.1%, 14.3%, and 16.3% of whom reacted to the relevant DCs, respectively.^118^ DCs with paracetamol, a weak COX-1 inhibitor, caused reactions in 11 (22.4%) of the 49 patients with N-ERD^118^ and in 3 (9.4%) of 32 patients with NIUAA.^116^ In the latter study,^116^ 3 (4.2%) of 72 patients with NIUAA reacted to etoricoxib DCs, whereas in another study,^117^ all 48 patients with NIUAA and all 34 with NECD tolerated celecoxib DCs.

Regarding paracetamol, the dose used for DCs is important.^16^^,^^123^^,^^124^ In a study,^123^ low doses of paracetamol (ie, 15 mg/kg) were tolerated by children aged 9–14 years with a well-demonstrated NIUAA. In another study^124^ of 7 children under 12 years of age who had reacted to ASA challenges and had tolerated challenges with the paracetamol cumulative dose of 80 mg, 2 reacted to the dose of 240 mg. These reactions may be related to the COX-1 inhibiting effects of paracetamol at high doses.

Importantly, reactions to DCs with alternative selective and preferential COX-2 inhibitors and paracetamol were milder than the index reactions.^16^^,^^53^ Nevertheless, even though reactions to DCs are generally easily controlled with symptomatic treatment, given the risk of cross-reactivity, a DC is still recommended to verify tolerability of an alternative agent prior to prescription for patients with NECD or NIUAA,^1^^,^^114^^,^^125^ and may be considered in patients with N-ERD and a history of anaphylaxis following NSAID exposure. DCs with alternative agents can be performed for example by administering one-tenth to one-quarter of the therapeutic dose followed by the remaining dose, with an observation period of 1–2 h after each of the two steps.^96^ Another suggested protocol might be a two-step administration of one-quarter and three-quarters of the therapeutic dose 1 h apart (eg, paracetamol 125 mg + 375 mg; nimesulide 25 mg + 75 mg).^126^ NECD patients should undergo DCs while they are not taking antihistamines. Of course, this is not always possible. In NECD patients, since the reaction generally occurs within a couple of hours^1^^,^^6^ and is clearly visible on clinical examination, the use of a placebo control can be skipped in the clinical practice. A positive test can be repeated with antihistamine premedication (eg, rupatadine 10 mg, cetirizine 10 mg, or desloratadine 5 mg, 30 min to 6 h before the test).^79^^,^^125^, ^126^, ^127^ In a study by Sánchez et al,^79^ the use of antihistamines prevented NSAID-exacerbated reactions in 75% of patients with CSU and allowed them to tolerate the NSAID involved in the previous reaction and even a strong COX-1 inhibitor.

## SNIUAA

### Clinical presentation

Some patients with NSAID hypersensitivity develop immediate reactions to a single NSAID or closely related drugs from the same chemical group, but tolerate other, non-chemically related NSAIDs (Table 2). The reaction is independent of the strength of COX-1 inhibition, pointing to a probable specific immunological mechanism.^1^ These reactions usually occur within the first hour following drug intake, although this interval can be longer (ie, up to 6 h), and symptoms may vary from skin reactions, such as urticaria/angioedema and generalized erythema, to anaphylaxis.^1^^,^^128^^,^^129^

In recent studies involving at least 30 adults diagnosed with NSAID hypersensitivity^4^^,^^9^^,^^18^^,^^23^^,^^24^^,^^130^ utilizing the classification system proposed by Kowalski et al,^1^ the rate of patients diagnosed with SNIUAA ranged from 15.9% (40 of 251 patients)^23^ to 47.2% (143 of 303)^4^ of those with a diagnosis of immediate NSAID hypersensitivity. The most involved NSAIDs were pyrazolones (ie, metamizole/dipyrone and propyphenazone) and propionic acid derivatives (ie, ibuprofen and ketoprofen).

In similar studies carried out in children and adolescents,^19^^,^^89^^,^^104^^,^^131^ the rate of patients with a diagnosis of SNIUAA ranged from 15.5% (16 of 103 patients)^89^ to 33.3% (11 of 33)^19^ of those diagnosed with immediate NSAID hypersensitivity. Paracetamol and ibuprofen were the most frequent elicitors of SNIUAA.

### Pathogenic mechanism

The pathogenic mechanism of SNIUAA is closely related to classical IgE-mediated allergic reactions to other drugs such as antibiotics.^1^^,^^129^ However, this has mainly been proven with pyrazolones.^132^ There may be a tendency for natural resolution of pyrazolone hypersensitivity over a period of years. Indeed, negativization of allergy tests has been observed in pyrazolone-allergic patients reevaluated more than 1 year after the first allergy examination.^133^, ^134^, ^135^

As with several drug hypersensitivity reactions, a specific genetic susceptibility may be a significant contributing factor.^136^

### Diagnosis

The diagnosis of SNIUAA is based on a history of well documented immediate urticarial/angioedematous/anaphylactic reactions to a single NSAID or to ≥2 NSAIDs belonging to the same chemical group in a patient without CSU. The diagnosis is firmly established if a patient has a history of tolerance of chemically unrelated NSAIDs taken after the reaction(s). Considering the pathogenic mechanism of SNIUAA, skin tests (STs) with the suspected NSAID may be helpful^1^^,^^15^ (Fig. 1). Table 3 shows the highest nonirritating NSAID concentrations for skin testing.^137^, ^138^, ^139^, ^140^ Several studies have demonstrated the usefulness of STs with pyrazolones, especially metamizole/dipyrone.^4^^,^^19^^,^^23^^,^^24^^,^^104^^,^^130^^,^^132^, ^133^, ^134^, ^135^^,^^141^^,^^142^ However, STs with NSAIDs other than pyrazolones (eg, diclofenac, ketorolac, and paracetamol) have rarely contributed to the diagnosis of immediate reactions.^4^^,^^19^^,^^143^, ^144^, ^145^, ^146^ Thus, STs cannot be recommended for most common NSAIDs, because they are not standardized,^6^ their negative predictive value has not yet been established, and some NSAIDs are not available for parenteral use.^147^ Regarding *in vitro* tests, such as serum specific IgE assay and BAT, they are not widely available and their diagnostic and predictive values also have yet to be validated.^147^, ^148^, ^149^ However, in patients with immediate reactions to metamizole, the BAT showed a sensitivity ranging from 42% to 65% and a specificity from 83% to 100%, proving to be complementary to STs.^102^ Therefore, DCs are often necessary to confirm or rule out a diagnosis of SNIUAA.^1^^,^^15^ However, DCs with the suspected NSAIDs are contraindicated in patients with severe anaphylaxis.^1^^,^^15^ Moreover, in a study by Li et al,^96^ a history of a recent reaction was associated with a higher rate of positive DCs. Therefore, the most effective diagnostic procedure in patients reporting SNIUAA is a graded DC with ASA, or with another potent COX-1 inhibitor like ibuprofen if ASA is the suspected drug.^1^^,^^82^^,^^86^^,^^92^^,^^104^^,^^105^^,^^150^ Indeed, if positive, this DC proves a pathogenic mechanism related to COX-1 inhibition and if negative confirms tolerance of a structurally unrelated agent, strongly supporting a diagnosis of SNIUAA without the need to expose the patient to the agent connected with an anaphylaxis event.^82^^,^^86^^,^^129^^,^^150^ Many studies have demonstrated the safety of this approach.^24^^,^^82^^,^^92^^,^^104^^,^^105^^,^^150^ Patients who report equivocal/doubtful and/or non-recent reactions are referred to a DC with ASA and, if tolerated, to DCs with the suspected NSAIDs (Fig. 1). It has been demonstrated that the total number of DCs per patient decreases if ASA DC is performed first, followed by additional DCs as needed.^104^^,^^105^ As previously mentioned, however, most European pediatric allergists do not perform ASA challenges to classify their patients with hypersensitivity reactions to NSAIDs.^107^

**Table 3:** Highest nonirritating concentrations of NSAIDs recommended for skin and patch testing

NSAIDs	Skin prick tests	Intradermal tests	Patch tests^a^	Photopatch tests
Acetylsalicylic acid			10% pet^b^	
Dexketoprofen				1% pet^197^
Ibuprofen			10% pet^b^ - 30%^d^,^198^	5% pet^197^
Ketoprofen	5 mg/mL^138^	5 mg/mL^138^	2.5% pet^b^- 30%^183^	1% pet^b^,^197^
Piketoprofen				1% pet^197^
Diclofenac	2.5 mg/mL^138^	2.5 mg/mL^138^	5% pet^b^	5% pet^b^,^197^
Ketorolac	1 mg/mL^143^	1 mg/mL^143^		
Etofenamate			30% pet^183^	2% pet^197^
Niflumic acid			Pure (gel)^198^	
Metamizole	400 mg/mL^15^^,^^137^	40 mg/mL^15^^,^^137^	10% water^137^ – pet^203^	
Other pyrazolones	0.1 mg/mL^139^	0.1 mg/mL^139^		
Piroxicam	2 mg/mL^140^	2 mg/mL^140^	1% pet^b^	1% pet^b^,^197^
Other injectable NSAIDs	0.1 mg/mL^139^	0.1 mg/mL^139^		
Paracetamol	10 mg/mL^15^	1 mg/mL^15^,^138^	10% pet^b^	
Celecoxib			30% pet^c^,^198^	

a In accordance with international guidelines,^199^^,^^200^ the test material obtained from trade products is used at concentrations of 10% or 30% in petrolatum (see text).

b Available as ready-to-use material (see text).

c 100-mg tablet.

d 400-mg tablet, diluted in water, petrolatum, or alcohol.

### Management

Patients with a confirmed diagnosis of SNIUAA must avoid the responsible drugs and those belonging to the same chemical group, because even the latter may induce reactions by cross-reactivity, as shown with propionic acid (eg, ibuprofen, ketoprofen, and flurbiprofen), acetic acid (eg, diclofenac and aceclofenac), and pyrazolone derivatives (eg, propyphenazone and metamizole/dipyrone).^151^^,^^152^

## NEFA/NIFA

### Clinical presentation

NSAIDs can act as exacerbating factors^153^, ^154^, ^155^, ^156^ or cofactors^153^^,^^156^, ^157^, ^158^, ^159^, ^160^, ^161^, ^162^, ^163^ of hypersensitivity reactions to food allergens, most of which are anaphylaxis. As exacerbating factors, NSAIDs aggravate hypersensitivity reactions to foods in individuals who have mild hypersensitivity reactions (e.g., oral allergy syndrome) to the foods concerned if ingested alone. When NSAIDs act as a cofactor, patients may fully tolerate a food if ingested alone, but experience a systemic reaction (eg, urticaria/angioedema, generalized erythema, anaphylaxis) after its ingestion in combination with NSAIDs.^23^^,^^24^ These phenomena have been termed FDNIH reactions.^23^ More recently, the former phenomenon has been termed NEFA and the latter NIFA^24^ (Table 2).

In individuals with food sensitization, exercise and alcohol also act as aggravating factors and/or cofactors, often in association with NSAIDs.^153^^,^^156^, ^157^, ^158^, ^159^, ^160^, ^161^, ^162^, ^163^, ^164^ An example is food-dependent exercise-induced anaphylaxis (FDEIA), a distinct form of food allergy characteristically induced by a combination of causative food ingestion and physical exercise, with NSAIDs acting as aggravating factors in some cases.^165^, ^166^, ^167^ Neither exercise nor food ingestion alone elicits this syndrome. FDEIA has also been reported in individuals taking low doses of ASA for reducing cardiovascular risk.^168^

### Pathogenic mechanism

It is likely that NEFA/NIFA and FDEIA share common pathophysiologic pathways. NSAIDs increase the absorption of allergens such as gliadin from the gastrointestinal tract, even in healthy control subjects,^169^, ^170^, ^171^ probably due to an impaired tight junction barrier secondary to diminished COX-1 dependent PG production in enterocytes.^171^ Long-lasting or high-intensity exercise can also alter intestinal permeability.^172^^,^^173^ It has been hypothesized that in some individuals, redistribution of blood flow during exercise might result in an ischemia/reperfusion cycle that causes epithelial damage,^174^ Moreover, NSAIDs and exercise have a direct effect on mast cells and basophils, amplifying their activation.^155^^,^^157^^,^^164^^,^^173^^,^^175^, ^176^, ^177^

The most frequently involved food allergens are ω-5 gliadin (Tri a 19)^156^^,^^158^^,^^160^^,^^164^^,^^167^ and non-specific lipid transfer proteins, mainly that of peach (Pru p 3).^23^^,^^24^^,^^153^, ^154^, ^155^^,^^162^^,^^163^^,^^166^^,^^178^^,^^179^ In a recent study,^162^ more than 30% of 528 adults sensitized to Pru p 3 had cofactor-related reactions, frequently severe. However, we should consider that there are remarkable geographic variations regarding the frequency of such sensitizations. For example, peach allergy is extremely uncommon in the USA.

### Diagnosis

Two studies prospectively evaluated patients with immediate reactions to NSAIDs, performing allergy tests to detect sensitization to food allergens and, in case of positive results, DCs with the suspected NSAIDs.^23^^,^^24^ In these studies,^23^^,^^24^ an FDNIH was diagnosed in 52 (15.9%) of 328 patients and NEFA/NIFA in 75 (18.1%) of 414 patients on the basis of: 1) mild reactions (i.e., oral allergy syndrome or contact urticaria) to (NEFA, 9 patients) or tolerance of (NIFA, 66 patients)^24^ the foods involved in the reaction—ascertained after the episode concerned—without taking NSAIDs; 2) systemic reaction (ie, urticaria, angioedema, generalized erythema, or anaphylaxis) in the context of food and NSAID intake (ie, maximum interval of 6 h between food ingestion and reaction and 8 h between NSAID intake and reaction,^23^ or after taking NSAIDs within 4 h before or after meals^24^); 3) positive allergy tests to food allergens involved in the reaction; and 4) negative DCs with the suspected NSAIDs. Pru p 3 was involved in 84.6% of 328 patients^23^ and 89.3% of 414 patients;^24^ Tri a 19 was involved in 11%^23^ and 6.7%^24^ of these patients, respectively.

Therefore, patients reporting immediate cutaneous and/or anaphylactic reactions to NSAIDs should be carefully questioned about the time interval between NSAID exposure and the last meal, as well as all foods ingested within 4 h before or after NSAID exposure (ie, suspected foods). They should undergo targeted allergy tests (ie, skin prick tests and serum specific IgE assays) with the main food allergens of the relevant geographic area. Patients presenting with positive allergy tests to the suspected foods should undergo DCs with the NSAIDs involved, avoiding the positive foods for 4 h before and after DCs (Fig. 1). Patients whose hypersensitivity to the culprit foods (ie, foods ingested within 4 h before or after NSAID exposure and found to be positive in the allergy workup) or whose tolerance of these foods has not yet been ascertained after the reactions should undergo open oral food challenges with the foods concerned.

### Management

Patients diagnosed with NEFA/NIFA should avoid NSAIDs, including ASA at anti-platelet doses, for 4 h before and after a meal containing foods that have been found to be positive in the allergy workup. This precaution allows all NEFA/NIFA patients to continue taking NSAIDs, including those involved in hypersensitivity reactions, without any reaction.^24^

## Single-NSAID-Induced Delayed Hypersensitivity Reactions (SNIDHRs)

### Clinical presentation

SNIDHRs are delayed immunologically mediated reactions^1^^,^^3^ characterized by different clinical manifestations that are mainly cutaneous (Table 4).^1^^,^^180^ The most frequent ones, such as fixed drug eruption (FDE), morbilliform drug eruption (MDE, also called maculopapular exanthem),^181^ contact dermatitis (CD), and photoallergic CD (PACD),^142^^,^^180^^,^^182^, ^183^, ^184^, ^185^ are mild/moderate. Less frequently, NSAIDs can induce severe cutaneous reactions, namely Stevens-Johnson syndrome/toxic epidermal necrolysis, drug reaction with eosinophilia and systemic symptoms/drug-induced hypersensitivity syndrome, and acute generalized exanthematous pustulosis (Table 4).^180^^,^^184^, ^185^, ^186^, ^187^, ^188^ Blood dyscrasias (eg, anemia, agranulocytosis, and thrombocytopenia)^142^^,^^185^^,^^189^ and organ-specific reactions (ie, drug-induced kidney injury, drug-induced liver injury, vanishing bile duct syndrome, drug-induced aseptic meningitis, and pneumonitis) have rarely been observed.^185^^,^^190^, ^191^, ^192^, ^193^

**Table 4:** *In* v*ivo* tests for evaluating delayed cutaneous reactions to NSAIDs

Reaction type	Main responsible NSAIDs^a^	Time interval^b^	Patch tests	dSPTs	dIDTs	Drug challenges
Morbilliform drug eruption	Metamizole;^142^ metamizole, ketoprofen^182^	4–14 days	Useful	Limited value	Useful	After negative skin tests in mild/moderate reactions
Contact dermatitis	Ketoprofen^183^	4 days-weeks	Useful	Unknown value	Unknown value	Unknown value
Systemic (allergic) contact dermatitis	Reports of <5 positive cases^205^^,^^209^	4 days-weeks	Useful	Limited value	Unknown value	After negative skin tests
SDRIFE	Reports of <5 positive cases^180^^,^^205^	Up to 7 days	Useful	Limited value	Useful	After negative skin tests
Photoallergic contact dermatitis	Ketoprofen, dexketoprofen, etofenamate;^204^ ketoprofen, etofenamate, piroxicam^210^	Days-years	Photopatch tests with low concentrations (1–5% in petrolatum) with a 5 J/cm^-2^ UVA irradiation	No value	No value	No value without exposure to UV
Fixed drug eruption^c^	Metamizole;^142^ metamizole, ketoprofen;^182^ piroxicam;^206^ mefenamic acid, piroxicam;^207^ etoricoxib^208^	30 min-8 h after readministration	Useful if applied on the area of eruption (see text)	Not useful	Not useful	At full dose when patch tests or repeated application tests are negative
GBFDE	Reports of <5 positive cases	4 days-weeks	May be useful	Contraindicated	Contraindicated	Contraindicated
AGEP	Reports of <5 positive cases^180^	1–12 days	Useful	Limited value	Potentially useful	Contraindicated with suspected drugs and cross-reactive ones
DRESS/DIHS	Reports of <5 positive cases^180^	2–8 weeks	Useful recommended 6 months after healing	Limited value	Reading at 24 h	Contraindicated with suspected drugs and cross-reactive ones
SJS/TEN	Reports of <5 positive cases^180^^,^^211^	4–28 days	Low sensitivity	Unknown value	Contraindicated with suspected drugs	Contraindicated with suspected drugs and cross-reactive ones
Vasculitis/SSLR		7–21 days	No value	No value	No value	Contraindicated

AGEP, acute generalized exanthematous pustulosis; DIHS, drug-induced hypersensitivity syndrome; dIDT, delayed-reading (after 24–48 h) intradermal test; DRESS, drug reaction with eosinophilia and systemic symptoms; dSPT, delayed-reading (after 24–48 h) skin prick test; GBFDE, generalized bullous fixed drug eruption; NSAID, non-steroidal anti-inflammatory drug; SDRIFE, symmetrical drug-related intertriginous and flexural exanthema; SSLR, serum sickness like reaction; SJS, Stevens–Johnson syndrome; TEN, toxic epidermal necrolysis.

a Found positive to patch tests and/or skin tests in at least 5 patients of the related studies performed in the last decade.

b Time interval between start of drug use and reaction. In repeated reactions, this time interval is generally shorter than that of the first reaction.^180^^,^^181^

c NSAIDs are one of the most common causes of fixed drug eruption globally.

The prevalence of SNIDHRs is not known. However, they represent the least common type of NSAID hypersensitivity in patients diagnosed by allergy testing.^4^^,^^96^^,^^130^

### Pathogenic mechanism

The pathogenic mechanism of SNIDHRs involves the stimulation of drug specific CD4^+^ and CD8^+^ T cells and does not differ from that of delayed reactions to other drugs.^1^^,^^194^^,^^195^ Cross-reactions between NSAIDs depend on the clinical features of the SNIDHR but are observed within the same chemical group of drugs.

### Diagnosis

**Fig. 2:**
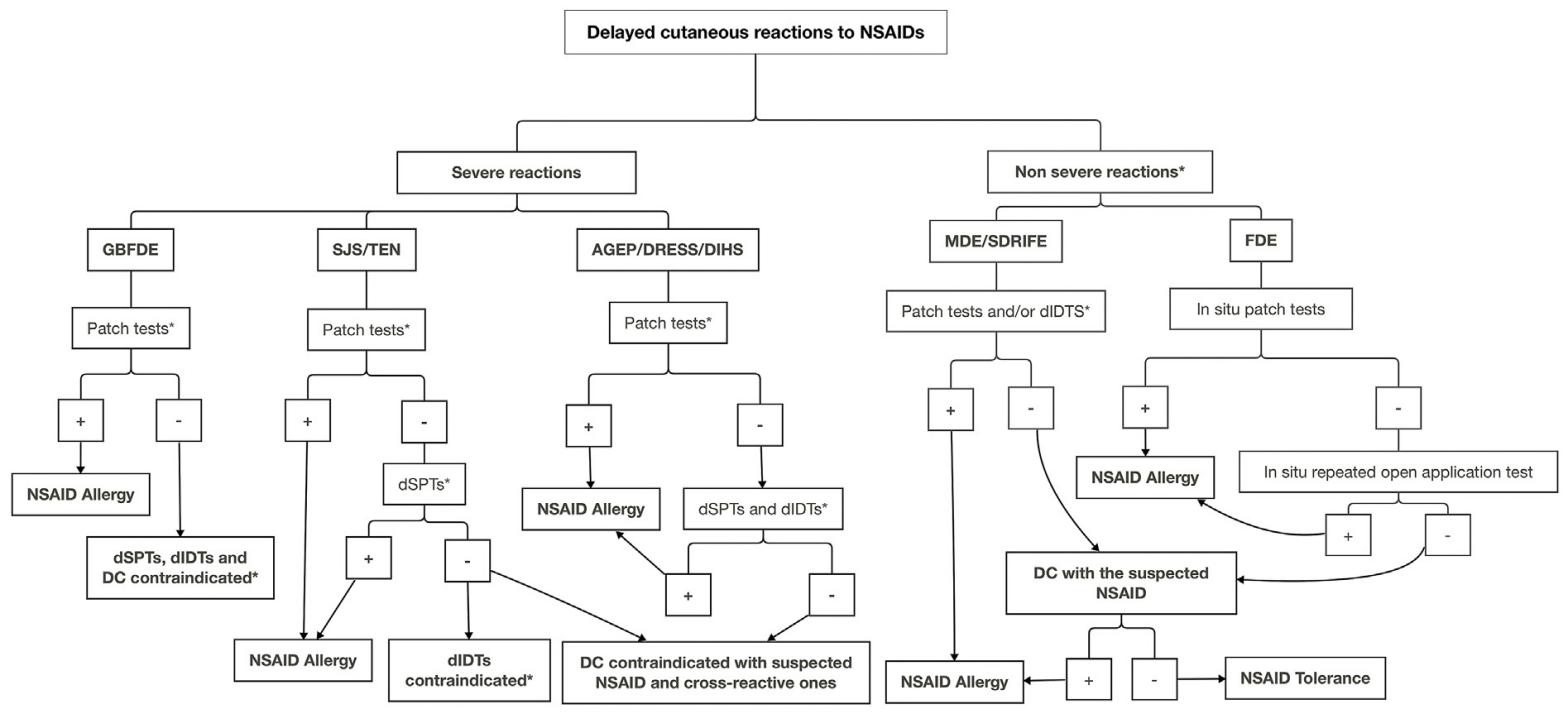
Diagnostic algorithm for patients with histories of delayed reactions to non-steroidal anti-inflammatory drugs AGEP, acute generalized exanthematous pustulosis; DC, drug challenge; dIDT, delayed-reading (after 24–48 h) intradermal test; DIHS, drug-induced hypersensitivity syndrome; dSPT, delayed-reading (after 24–48 h) skin prick test; DRESS, drug reaction with eosinophilia and systemic symptoms; FDE, fixed drug eruption; GBFDE, generalized bullous fixed drug eruption; MDE, morbilliform drug eruption; NSAID, non-steroidal anti-inflammatory drug; SDRIFE, symmetrical drug-related intertriginous and flexural exanthema; SJS, Stevens-Johnson syndrome; TEN, toxic epidermal necrolysis. ∗See Table 4

The diagnosis is mainly based on clinical history and *in vivo* tests selected according to the type of reaction (Table 4 and Fig. 2). Patch tests (PTs) and STs, namely skin prick tests and delayed-reading intradermal tests (dIDTs) have been used to identify the responsible NSAID, as well as to assess cross-reactivity.^140^^,^^194^^,^^196^ Table 3 shows the highest nonirritating concentrations of NSAIDs recommended for STs and PTs, according to literature data.^137^, ^138^, ^139^, ^140^^,^^197^^,^^198^ However, concentrations for STs were defined mainly regarding immediate reactions. For intradermal testing, the highest nonirritating concentration of many NSAIDs might be lower than that which evokes a T-cell response after 6–24 h.^140^^,^^194^ In systemic reactions to NSAIDs, for patch testing, unfortunately only a limited number of molecules marketed by Chemotechnique (Velinge, Sweden) and SmartPractice Europe GmbH (Greven, Germany) are available as ready-to-use material at concentrations ranging from 1% to 10% in petrolatum (Table 3). In accordance with international guidelines,^199^^,^^200^ the material for testing NSAIDs not available as ready-to-use reagents has to be prepared in-house from the drugs used by the patients. When the pure active NSAID is available, from the powder of an injectable preparation or from capsules, it is preferred to tablets for preparing material for patch testing. After being ground to a fine powder, the material should be incorporated in petrolatum, whenever possible to have the active principle in a final 10% (wt/wt) dilution. When the concentration of the active drug is too low in the patient's drug, the whole powder should be diluted in petrolatum at 30%.^139^^,^^140^^,^^183^^,^^196^^,^^198^, ^199^, ^200^ PTs and dIDTs have a high specificity, but a low sensitivity;^140^^,^^194^ dIDTs with metamizole are more sensitive than PTs.^137^ In FDE, it is crucial to apply PTs on the site of eruption (residual sometimes pigmented lesion; ie, “*in situ* PTs”).^140^^,^^194^^,^^201^^,^^202^ If *in situ* PTs are negative, an *in situ* repeated open application test can be done.^140^^,^^194^^,^^202^ Positive responses to PTs (and/or dIDTs with metamizole) have been observed mainly in FDE, CD, PACD, MDE, systemic (allergic) contact dermatitis, and symmetrical drug-related intertriginous flexural exanthema,^141^^,^^142^^,^^180^^,^^182^, ^183^, ^184^, ^185^^,^^201^^,^^203^, ^204^, ^205^, ^206^, ^207^, ^208^, ^209^, ^210^ but also in severe cutaneous reactions^137^^,^^180^^,^^203^^,^^211^ and drug-induced aseptic meningitis.^193^

In mild/moderate reactions, such as MDE and FDE, negative STs and/or PTs can be followed by a DC. There is no standardized protocol for DCs in delayed reactions to drugs.^194^ As for other drugs, a recent European position paper recommends reaching the maximum single therapeutic/unit dose in one day and in 1–3 steps, depending on the severity of the MDE.^212^ A more cautious protocol, which simplifies the one of the study of Viola et al,^138^ consists in administering initially one-tenth of the therapeutic dose and, if tolerated, a full dose 1–7 days after, depending on the time interval of the index reaction. If the patient requires therapy urgently, the one-tenth dose (if tolerated) should be followed 1 h later by the remaining dose. Regarding *in vitro* methods, lymphocyte transformation test and enzyme-linked immunosorbent spot assay have been used for identifying the culprit drugs, including NSAIDs, in patients with delayed reactions, especially severe cutaneous ones. However, expert consensus on the diagnostic value of these tests has not yet been reached.^148^

### Management

Patients with a confirmed diagnosis of delayed hypersensitivity to NSAIDs must avoid the responsible drugs and prudently also those belonging to the same chemical group. Cross-reactivity has been mainly observed among oxicams in patients with FDE,^201^^,^^206^^,^^213^ but also among the components of each of the other chemical groups (eg, acetic acid derivatives, propionic acid derivatives, and pyrazolones) in individuals with different types of SNIDHRs, including FDE.^213^^,^^214^ Patients must also be informed about alternative NSAIDs. Cross-reactions have specific mechanisms in NSAID-induced PACD.

Ketoprofen photoallergies are due to the chemical benzophenone structure. This structure can be found in other drugs such as fenofibrate and tiaprofenic acid, and in many chemical sunscreens such as oxybenzone or mexenone. In cases of photosensitivity to ketoprofen, all the above drugs and chemical sunscreens should be avoided both topically and systemically. The use of high sun protection factor (SPF) mineral sunscreens is recommended to prevent photo-remanence.^215^

The sensitizing photometabolite of piroxicam is thiosalicylic acid. Cross-allergy is constant with thiomersal, which is partly composed of thiosalicylic acid. Thiomersal, a mercurial preservative, can still be found in certain eye drops. In piroxicam photoallergy, other oxicams are well tolerated.^216^ Patients with photosensitization to diclofenac cross react with aceclofenac.^214^

## Conclusions

This WAO Statement updated both the classification of hypersensitivity reactions to NSAIDs (Table 2) and their diagnosis. Regarding the former, patients with no underlying CSU who report reactions to ≥2 chemically unrelated NSAIDs that involve urticaria/angioedema and/or 2 organ systems (e.g., cutaneous and respiratory; cutaneous and gastrointestinal) were classified as having NIUAA. Patients with N-ERD who also report cutaneous symptoms and patients with NECD who report respiratory symptoms following NSAID intake were diagnosed as having mixed N-ERD and mixed NECD, respectively. These reactions could also be called anaphylactic reactions in patients with NECD or N-ERD. Furthermore, reactions in which NSAIDs act as aggravating factors or cofactors in subjects with sensitization to foods were included and defined as NEFA and NIFA, respectively. In the updated diagnostic algorithm, special account was taken of these reactions.

An updated and correct classification of hypersensitivity reactions to NSAIDs has important repercussions on the diagnostic workup and management of patients who report such reactions, with a positive impact on the healthcare delivery and patient outcomes, especially for those in whom hypersensitivity to NSAIDs is excluded.

## Author contributions

Romano (Chair of the Task Force) and Valluzzi (Co-Chair of the Task Force) contributed to conceiving, designing, editing, and revising the manuscript. All authors performed the literature research and contributed to the drafting of the respective topics. Specifically, Cahill, Makowska, Picado, Sanak, and Taniguchi co-authored N-ERD topic, which was coordinated by Cahill and Picado. Asero and Bavbek co-authored NECD topic; Doña, Li, and White co-authored NIUAA topic; Demoly and Kidon co-authored SNIUAA topic. NECD, NIUAA, and SNIUAA topics were coordinated by Asero, Kidon, and White. Bartra, Guzmán Meléndez, Romano, and Valluzzi co-authored NEFA/NIFA topic, which was coordinated by Bartra. Barbaud, Park, and Romano co-authored SNIDHR topic, which was coordinated by Barbaud and Park. The Steering Committee Authors are in alphabetical order (except for the chair and the co-chair of the task force). The Review Panel Members are in alphabetical order.

## Ethics approval

Ethics approval not applicable.

## Consent for publication

All authors gave their consent to publish this work.

## Availability of data and material

Not applicable.

## Funding

The World Allergy Organization provided funding for the open-access publication of this article. Otherwise, there was no other source of funding for this article.

## Declaration of competing interest

The authors report no competing interests concerning this document.
